# Sleep duration, sleep problems and developmental trajectories of urinary incontinence: a prospective cohort study

**DOI:** 10.1007/s00787-024-02471-1

**Published:** 2024-06-03

**Authors:** Carol Joinson, Mariusz T. Grzeda, Jon Heron, Alexander von Gontard

**Affiliations:** 1 Centre for Academic Child Health, Population Health Sciences, Bristol Medical School, University of Bristol, Canynge Hall, 39 Whatley Road, Bristol, BS8 2PS UK; 2 Galen Research, B1 Chorlton Mill, 3 Cambridge Street, Manchester, M1 5BY UK; 3 Population Health Sciences, Bristol Medical School, University of Bristol, Oakfield House, Oakfield Grove, Bristol, BS8 2BN UK; 4 Psychiatric Services Graubünden (PDGR), Outpatient Services for Child and Adolescent Psychiatry, Chur, Switzerland; 5 Governor Kremers Centre, Department of Urology, Maastricht University Medical Centre, Maastricht, The Netherlands

**Keywords:** Sleep problems, Urinary incontinence, Enuresis, Child development, Prospective cohort study, ALSPAC

## Abstract

To examine if preschool sleep duration and sleep problems are associated with urinary incontinence (UI) at primary school-age. We used multinomial logistic regression to examine the association of child sleep duration/problems (3½ years) with UI trajectories (4–9 years) in 8751 (4507 boys, 4244 girls) from the Avon Longitudinal Study of Parents and Children. We adjusted for sex, socioeconomic indicators, mothers’ emotional/practical/financial support, developmental delay, stressful life events, temperament, and emotional/behaviour problems. Preschool children who slept more than 8½ hours per night had a decreased probability of UI at school-age. There was a 33% reduction in odds of daytime wetting per additional hour of sleep (odds ratio [OR] = 0.67, 95% confidence interval [CI] 0.52–0.86). Sleep problems were associated with increased odds of UI e.g., getting up after being put to bed was associated with daytime wetting (OR = 2.20, 95% CI 1.43–3.39); breathing problems whilst sleeping were associated with delayed bladder control (OR = 1.68, 95% CI 1.12–2.52), and night-time waking was associated with persistent (day and night) wetting (OR = 1.53, 95% CI 1.16–2.00). Waking during the night and waking up early in the morning were associated with reduced odds of bedwetting at school-age (OR = 0.76, 95% CI 0.61–0.96 and OR = 0.80, 95% CI 0.64–0.99 respectively). Preschool children who sleep for longer have a lower likelihood of UI at school-age, whilst those with sleep problems are more likely to experience daytime wetting and combined (day and night) wetting, but not bedwetting alone. Short sleep duration and sleep problems in early childhood could be indicators of future problems attaining and maintaining bladder control.

## Introduction

Sleep is important for a wide range of paediatric health outcomes and there is growing evidence that sleep problems in early childhood have possible long-term consequences for child and adolescent psychosocial and physical health [1]. A shorter night-time sleep duration, lack of a regular sleep pattern, and sleep problems such as night-time waking are associated with adverse developmental consequences for socio-emotional and cognitive development and poor health outcomes including overweight/obesity [2].

Acquisition of bladder control is a key developmental milestone that has been examined in relation to sleep. Most of the existing research focuses on bedwetting (enuresis) and comprises small case–control studies [3, 4]. This previous work implicates sleep problems in the pathophysiology of bedwetting. Compared with children who are dry at night, children with bedwetting have more sleep problems (e.g. bedtime resistance, longer duration of sleep latency, difficulty falling asleep, shorter duration of continuous sleep, parasomnias, sleep-disordered breathing, night-time waking) [5–8]. A prospective cohort study examined factors associated with nocturnal bladder control, including duration of sleep in children aged 1–2 years, and found that children with a longer sleep duration were slower to attain nocturnal bladder control [9]. The study found that 3.3% of children were reported by mothers to have attained nocturnal bladder control by age eight years, but the prevalence of bedwetting at age eight was 7.4%. This was due to some children relapsing following the attainment of nocturnal bladder control [9].

The association between sleep problems and both bedwetting and daytime wetting was previously examined in a cross-sectional study (a community-based study of 638 children with mean age 5.9 years) [10]. The study found that sleep problems were not more common in children with bedwetting and/or daytime wetting compared with continent children. Contrary to earlier research, children with enuresis had fewer night-time awakenings than their peers who had attained night-time bladder control [10].

No previous studies examined whether sleep problems are differentially associated with bedwetting alone versus bedwetting that is accompanied by daytime wetting. This is important because these different patterns of urinary incontinence (UI) are thought to have different underlying pathophysiology [11]. Specifically, bedwetting that is accompanied by daytime lower urinary tract symptoms (LUTS) including daytime wetting is defined as non-monosymptomatic enuresis, whilst monosymptomatic refers to bedwetting that occurs in the absence of daytime LUTS. Another limitation is that none of the studies adjusted for common risk factors for sleep problems and incontinence to assess whether observed associations are due to confounding.

The current study, based on data from a large birth cohort, examines prospective relationships between sleep duration and sleep problems in children at 3½ years and different patterns of UI at primary school-age (4–9 years) including normative development of bladder control, delayed attainment of bladder control (day and night), bedwetting alone, daytime wetting alone, and persistent (day and night) wetting. Data were available on a range of maternally reported sleep problems in young children including refusal to go to bed, difficulty going to sleep, getting up after being put to bed, getting up after little sleep, night-time waking, breathing problems while asleep, snoring, nightmares, waking up early, and lack of a regular sleep pattern.

## Methods

### Participants

Data were obtained from the Avon Longitudinal Study of Parents and Children (ALSPAC)—a large UK-based prospective birth cohort. Pregnant women resident in Avon, UK with expected dates of delivery between 1st April 1991 and 31st December 1992 were invited to take part. The initial number of pregnancies enrolled was 14,541. Of the initial pregnancies, there was a total of 14,676 foetuses, resulting in 14,062 live births and 13,988 children who were alive at 1 year of age. When the oldest children were approximately 7 years of age, an attempt was made to bolster the initial sample with eligible cases who had failed to join the study originally. As a result, when considering variables collected from the age of seven onwards (and potentially abstracted from obstetric notes) there are data available for more than the 14,541 pregnancies. The total sample size for analyses using any data collected after the age of seven is therefore 15,447 pregnancies, resulting in 15,658 foetuses. Of these 14,901 children were alive at 1 year of age [12, 13]. The study website contains details of all data that are available through a fully searchable data dictionary and variable search tool (http://www.bristol.ac.uk/alspac/researchers/our-data/).

Ethical approval was obtained from the ALSPAC Ethics and Law Committee and the Local Research Ethics Committee. Full details of the Research Ethics Committee approval references can be accessed here: https://www.bristol.ac.uk/media-library/sites/alspac/documents/governance/Research_Ethics_Committee_approval_references.pdf. Informed consent for use of data collected via questionnaires and clinics was obtained from participants following the recommendations of the ALSPAC Ethics and Law Committee at the time.

### Exposures: sleep duration and sleep problems at 3½ years

#### Night-time sleep duration

Mothers were asked about the time in the evening when their child normally goes to sleep and the time when their child normally wakes. They were asked to indicate the time using the 24-h clock. Total night-time sleep duration was calculated as the difference between time of waking and time of going to bed.

#### Sleep problems

Mothers were also asked whether their child has a regular sleep routine (*yes, no*) from which we derived a binary variable (‘1’ = no regular sleep routine). Variables for other sleep problems were derived from questions with more than two categories, which we dichotomized to indicate if the problem was present (at any level) or not e.g., night-time waking was derived from the number of times the child wakes up at night (‘0’ = no waking up and ‘1’ = waking one or more times). Variables for specific sleep problems (refusal to go to bed, difficulty going to sleep, getting up after being put to bed, getting up after little sleep, waking up at night, getting up after little sleep, waking up early, nightmares, waking up early, and lack of a regular sleep pattern) had options: ‘*No, did not happen’, ‘Yes, but didn’t worry me’*, *‘Yes, worried me a bit’, ‘Yes, worried me greatly’.* We coded the first option as ‘0’ and all remaining options as ‘1’. The question on snoring had the options *‘only rarely’*, *‘quite often’* and *‘most nights’* and the question on breathing problems while asleep had the options *‘no’*, *‘yes’, sometimes’* and *‘yes, often’*. For these questions, we coded the first option as ‘0’ and all remaining options as ‘1’.

### Outcomes: developmental trajectories of urinary incontinence at 4–9 years

At ages 4^1^/_2_, 5^1^/_2_, 6^1^/_2_, 7^1^/_2_ and 9^1^/_2_ years (hereafter referred to as 4–9 years) parents were asked “How often does your child wet him/herself during the day?” and “during the night?” and both questions were given the response options ‘Never’; ‘occasional accidents but less than once a week’; ‘About once a week’; ‘2–5 times a week’; ‘Nearly every day’; and ‘More than once a day’. In an earlier study, we applied longitudinal latent class analysis (LLCA) to these data to extract five longitudinal latent classes (developmental trajectories) describing different patterns of UI at 4–9 years [14]. The *normative* class had the highest prevalence (63.0% of the sample) and was characterized by a very low probability of daytime wetting or bedwetting at 4 years. There were four atypical classes describing different patterns of childhood UI: *delayed attainment of bladder control* (decreasing probability of bedwetting from 6 to 9 years with accompanying daytime wetting—8.6% of the sample); *bedwetting alone* (15.6%); *daytime wetting alone* (5.8%), and *persistent wetting* (persistent bedwetting to age 9 with accompanying daytime wetting) (7.0%). The proportion of boys in the *normative* class was 47.5%. A higher percentage of boys than girls belonged to the *bedwetting alone* (68.5%) and *persistent wetting* (63.1%) classes, whilst the *daytime wetting alone* class had a lower percentage of boys (33.8%). A slightly higher proportion of boys were in the *delayed* class (52.5%).

### Confounders

Models were adjusted for child’s sex and indicators of parental socioeconomic background including occupational social class (non-manual vs. manual); housing adequacy (based on information on crowding, periods of homelessness, living conditions; major defects/infestation); maternal education (none/vocational, O-level [UK exams taken at 16 years], A-level [UK high-school exams taken at 18 years] and above); presence of mother’s major financial problems (binary variable), and presence of mother’s social network (emotional/practical/financial support).

Children’s developmental level at 18 months was assessed using a parent-reported questionnaire developed by ALSPAC including items from the Denver Developmental Screening Test [15]. We used a total development score adjusted for age in weeks, standardized (using a linear regression model and extracting the residuals), and reversed where appropriate so that high values on all scores reflected a lower level of development.

Stressful life-events were assessed at 3½ years using the question, “*Below are listed some events that might upset some children. Please state whether any of these happened in the past 12 months?*” We generated a total life-events score by adding all events (presence of life-event = 1, absence = 0) and then standardized the score [16].

Mothers completed the Toddler Temperament Scale (TTS) at 2 years [17] and we restricted our analysis to adjusting for five traits (activity, adaptability, intensity, mood, persistence) that were associated with incontinence in an earlier study [18]. Child temperament was also assessed at 3 years using the Emotional Activity Sociability questionnaire [19] comprising four subscales (emotionality, activity, shyness, and sociability).

Psychological problems were assessed using the Revised Rutter Parent Scale [20] administered at 3½ years and comprising five subscales (emotional difficulties, conduct difficulties, hyperactivity, prosocial behavior, and total behavioral difficulties).

### Statistical analysis

We estimated the prospective association between sleep duration/sleep problems and the UI classes using a series of multinomial logistic regression models and employing the normative class as the reference category. We obtained parameter estimates using the “Modal ML” 3-step method [21] implemented in Mplus with the “auxiliary (r3step)” command. This approach has been shown to produce less-biased estimates than traditional three-step methods such as standard probability weighting or modal assignment, whilst avoiding the problem of covariates impacting on the measurement model itself [22].

## Results

The mean night-time sleep duration at 3½ years was 11.5 h (SD = 1.1 h, range = 6 h to 16.5 h) and the most common sleep problems were waking up early, waking at night, refusal to go to bed, difficulty going to sleep, getting up after being put to bed, and nightmares (Table 1).

**Table 1:** Sleep duration and prevalence of sleep problems in children at 3½ years

Sleep duration	Mean (SD)
Mean night-time sleep duration in hours (SD)	11.5 (1.1)
Sleep problems^1^	*N* (%)
Refusal to go to bed	3454 (41.8%)
Difficulty going to sleep	3029 (36.6%)
Gets up after being put to bed	2958 (35.8%)
Gets up after little sleep	1334 (16.1%)
Wakes at night	3558 (43.4%)
Breathing problems while asleep	1066 (14.8%)
Snores	1558 (20.7%)
Nightmares	2712 (32.8%)
Wakes up very early	4456 (53.9%)
Lack of regular sleeping pattern	602 (7.3%)

^1Total*N*for each type of sleep problem varies according to availability of data^

### Association between night-time sleep duration at 3½ years and the UI classes at 4–9 years

Table 2 shows the unadjusted and adjusted odds ratios and 95% confidence intervals for the association between night-time sleep duration at 3½ years and the UI classes at 4–9 years. A longer night-time sleep duration at 3½ years was associated with a 33% (14% to 48%) reduction in the odds of membership of the ‘daytime wetting alone’ class per additional hour of sleep. There was weaker evidence that a longer night-time sleep duration was associated with decreased odds of membership of the ‘delayed’ and ‘persistent wetting’ classes.

**Table 2:** Odds ratios and 95% confidence intervals for the association between sleep duration and sleep problems at 3½ years and patterns of urinary incontinence at 4–9 years

	Bedwetting alone	Daytime wetting alone	Delayed	Persistent wetting	*p* value
Sleep duration (hours)					
Unadjusted	0.88 [0.79, 0.97]	0.79 [0.66, 0.93]	0.78 [0.68, 0.90]	0.82 [0.72, 0.92]	< 0.001
Adjusted	0.92 [0.81–1.06]	0.67 [0.52–0.86]	0.85 [0.72–1.01]	0.87 [0.74–1.01]	0.003
Refusal to go to bed					
Unadjusted	1.05 [0.88, 1.24]	1.19 [0.87, 1.62]	1.29 [1.03, 1.63]	0.99 [0.80, 1.22]	0.160
Adjusted	1.03 [0.82–1.29]	1.29 [0.85–1.98]	1.22 [0.91–1.63]	0.72 [0.54–0.96]	0.058
Difficulty going to sleep					
Unadjusted	1.08 [0.91, 1.29]	1.84 [1.35, 2.50]	1.22, [0.96, 1.55]	1.34 [1.08, 1.66]	< 0.001
Adjusted	1.06 [0.84–1.33]	1.86 [1.22–2.83]	1.27 [0.93–1.72]	1.16 [0.88–1.54]	0.015
Gets up after being put to bed					
Unadjusted	1.00 [0.83, 1.12]	1.66 [1.22, 2.27]	1.33 [1.05, 1.69]	1.65 [1.33, 2.04]	< 0.001
Adjusted	1.03 [0.82–1.30]	2.20 [1.43–3.39]	1.13 [0.84–1.53]	1.22 [0.92–1.62]	0.004
Gets up after little sleep					
Unadjusted	1.00 [0.79, 1.27]	1.19 [0.79, 1.78]	1.32 [0.98, 1.77]	1.32 [1.01, 1.72]	0.070
Adjusted	0.94 [0.68–1.29]	1.20 [0.68–2.10]	1.22 [0.84–1.77]	1.04 [0.72–1.50]	0.769
Night-time waking					
Unadjusted	0.96 [0.81, 1.14]	1.19 [0.87, 1.62]	1.42 [1.12, 1.79]	1.41 [1.14, 1.74]	< 0.001
Adjusted	0.76 [0.61–0.96]	1.12 [0.74–1.70]	1.35 [1.01–1.80]	1.53 [1.16–2.00]	< 0.001
Breathing problems while asleep					
Unadjusted	1.15 [0.89, 1.49]	1.50 [0.98, 2.31]	1.45 [1.05, 2.01]	1.27 [0.94, 1.72]	0.019
Adjusted	1.07 [0.76–1.51]	1.42 [0.79–2.59]	1.68 [1.12–2.52]	1.17 [0.78–1.77]	0.069
Snores					
Unadjusted	1.11 [0.89, 1.38]	0.97 [0.65, 1.48]	1.27 [0.95, 1.70]	1.27 [0.98, 1.64]	0.114
Adjusted	1.22 [0.92–1.62]	0.66 [0.35–1.24]	0.97 [0.65–1.45]	1.13 [0.80–1.59]	0.275
Nightmares					
Unadjusted	1.16 [0.97, 1.39]	1.34 [0.98, 1.85]	1.33 [1.05, 1.70]	1.14 [0.91, 1.43]	0.013
Adjusted	1.11 [0.88–1.40]	1.28 [0.83–1.98]	1.21 [0.89–1.64]	1.04 [0.78–1.39]	0.477
Wakes up early					
Unadjusted	0.86 [0.73, 1.02]	0.92 [0.67, 1.25]	1.34 [1.05, 1.70]	1.17 [0.94, 1.44]	0.026
Adjusted	0.80 [0.64–0.99]	0.80 [0.53–1.21]	1.32 [0.98–1.78]	1.05 [0.80–1.38]	0.078
Lack of regular sleep pattern					
Unadjusted	0.93 [0.66, 1.31]	1.46 [0.87, 2.46]	1.43 [0.97, 2.10]	0.76 [0.47, 1.22]	0.105
Adjusted	0.75 [0.43–1.28]	1.71 [0.88–3.33]	1.38 [0.77–2.48]	0.55 [0.27–1.14]	0.074

Odds-ratios presented are derived in the relation to the normative class

Models were adjusted for: child’s sex; parental social class (non-manual vs. manual); housing adequacy index (based on information on crowding, periods of homelessness, living conditions; major defects/infestation); maternal education (defined by three categories: none/vocational, O-level, A-level or more); presence of mother’s major financial problems (binary variable); presence of social network including a) emotional support b) practical/financial support; standardized score on the child developmental level test (measured according to Denver Developmental Screening Test at 18 months); standardized score of stressful life events (at 42 months); child temperament scales derived from Toddler Temperament Scales at 24 months (activity, adaptability, intensity, mood persistence); temperament derived from Emotional Activity Sociability questionnaire: emotionality, activity level; shyness; sociability (at 42 months); Revised Rutter Parent Scales: emotional difficulties; conduct difficulties; hyperactivity; prosocial behavior (at 42 months)

**Fig. 1:**
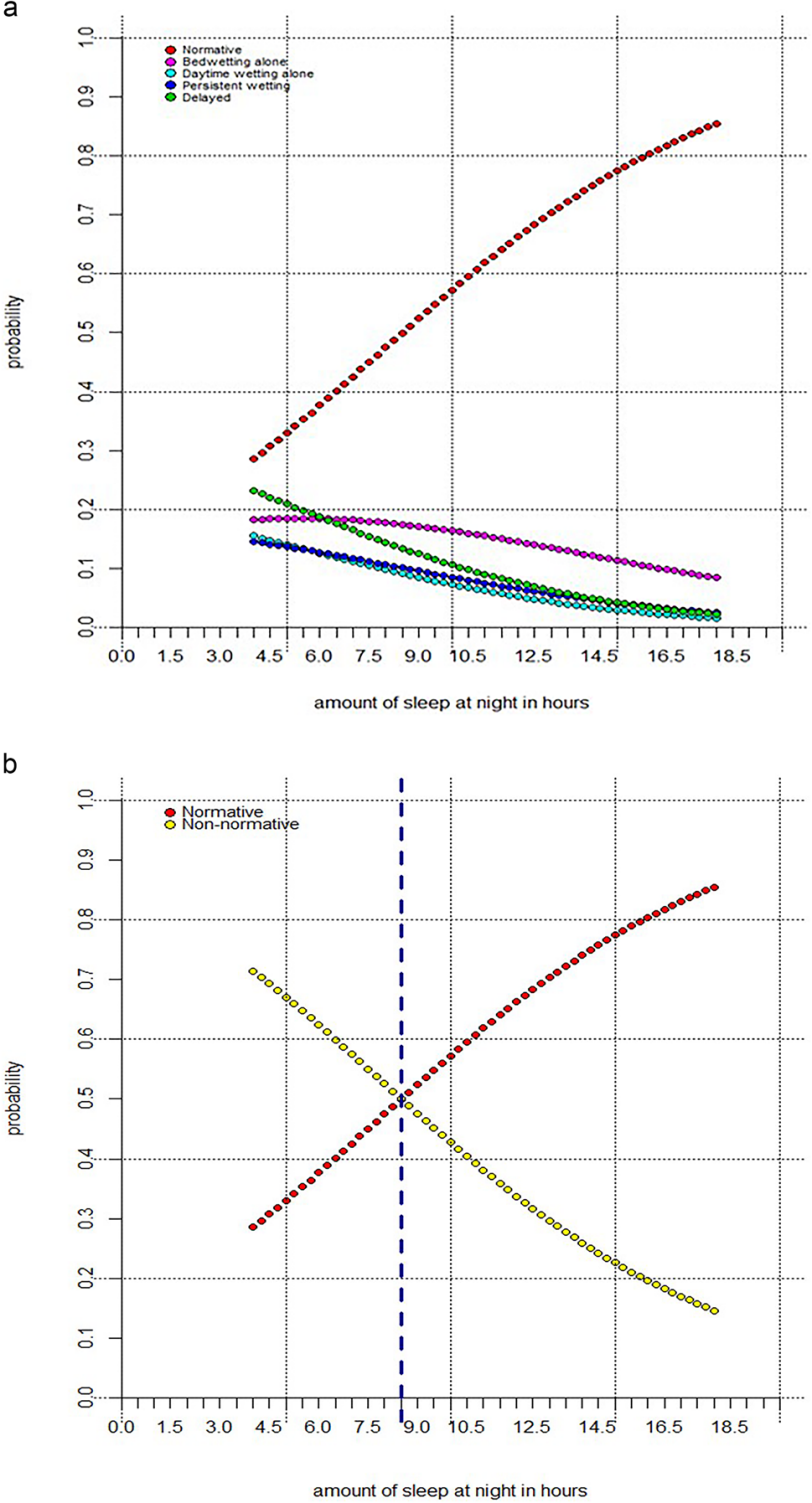
**a** Probability of being in a particular urinary incontinence class at 4–9 years as a function of night-time sleep duration at 3½ years. **b** Probability of being in the normative versus any atypical (non-normative) class at 4–9 years as a function of night-time sleep duration at 3½ years

Fig. 1a shows the probability of being in each UI class at 4–9 years in relation to night-time sleep duration at 3½ years. The probability of being in one of the atypical UI classes decreased with every additional hour of sleep. When all atypical classes were aggregated (Fig. 1b) the results show that the probability of membership to an atypical (i.e., non-normative) UI class increases if the amount of night-time sleep falls below a threshold of 8½ hours at 3½ years.

### Association between sleep problems at 3½ years and the UI classes at 4–9 years

Table 2 shows the unadjusted and adjusted odds ratios and 95% confidence intervals for the association between sleep problems at 3½ years and the UI classes at 4–9 years. Sleep problems including difficulty going to sleep and getting up after being put to bed were strongly associated with increased odds of membership of the ‘daytime wetting alone’ class. Night-time waking was associated with an increase in the odds of membership of the ‘delayed’ and ‘persistent wetting’ classes. Breathing problems whilst sleeping were associated with an increase in the odds of membership of the ‘delayed class. There was weak evidence of an association between waking up early and increased odds of membership of the delayed class.

Night-time waking and waking up early were associated with decreased odds of membership of the ‘bedwetting alone’ class. Refusal to go to bed was associated with reduced odds of membership of the ‘persistent wetting’ class. Lack of a regular sleep pattern, snoring, refusal to go to bed, nightmares, and getting up after little sleep were not associated with membership of the UI classes.

## Discussion

### Main findings

The current study is unique because it examines long-term associations between sleep duration and sleep problems in early childhood and developmental trajectories of UI. Children who had a longer night-time sleep duration at 3½ years were less likely to suffer from UI, especially daytime wetting, at school-age. Preschool children who slept more than 8½ hours per night had a decreased probability of experiencing any type of UI at school-age. Sleep problems in preschool children were associated with daytime wetting alone (difficulty going to sleep, getting up after being put to bed), delayed attainment of daytime and night-time bladder control (night-time waking, breathing problems whilst sleeping), and persistent day and night wetting (night-time waking). Sleep problems were not more common in children with bedwetting alone. Instead, night-time waking and waking up early at age 3½ years were associated with a lower likelihood of children wetting the bed at primary school-age. We found that refusal to go to bed was associated with reduced odds of persistent wetting. This was an unexpected finding that could have been due to chance. It is notable that refusal to go to bed was one of the most common sleep problems in 3½ year-olds in the ALSPAC cohort, which is consistent with other studies [23].

### Strengths and limitations

Key strengths of this study include the prospective design based on a large community-based cohort, the availability of data on sleep duration and sleep problems in preschool children, and the use of developmental trajectories of UI from the age of 4–9 years (rather than measurement of incontinence at a single timepoint). We adjusted for a wide range of confounders which have previously been found to have robust associations with sleep problems and UI in children. We have not based our conclusions purely on p value thresholds (e.g. p < 0.05) to determine statistical significance, but instead, we consider the effect estimates alongside the strength of evidence indicated by the p values and confidence intervals in line with current recommendations [24, 25].

We did not restrict our analysis to children who met clinical diagnostic criteria for bedwetting or daytime wetting, therefore, the study provides evidence that sleep duration/problems are associated with UI in a non-clinical sample of children in the community. The prospective cohort design reduces the possibility of reverse causality and recall bias as alternative explanations for our findings. This is important because it has been suggested that parents of enuretic children are more likely than those of continent children to check their child during sleep and, therefore, may be more aware of their child’s sleep problems [6]. This is unlikely in our study because parents provided assessments of their child’s sleep problems before an age at which bedwetting would typically be viewed as abnormal.

Whilst most previous studies focused on sleep and enuresis [3, 4], a strength of our study is the inclusion of daytime wetting, which has been a neglected topic in existing research. A limitation is that the ‘bedwetting alone’ class did not exclude children with other lower urinary tract symptoms such as urgency and frequency irrespective of daytime wetting. Consequently, we were unable to fully ascertain if there are differential associations with monosymptomatic versus non-monosymptomatic enuresis [11]. Another limitation is the reliance on parental reports of sleep problems rather than clinical assessment of sleep disorders. Polysomnography is the gold standard for the assessment of sleep disorders, but this was not feasible in a large community-based birth cohort study. Also, we are unable to generalise our results to minority ethnic groups and less affluent populations because the ALSPAC cohort is predominantly white and affluent.

### Comparison with previous findings

Previous studies have focused on the comorbidity (co-occurrence at the same time point) of sleep problems and enuresis. Only one previous cohort study examined prospective associations and found that a longer sleep duration in infancy was associated with delayed attainment of night-time bladder control [9]. There are several possible reasons for the difference in findings. The previous study examined only night-time bladder control as the outcome whilst we examined different patterns of bedwetting and daytime wetting. They assessed sleep duration as the typical length of time the child slept throughout a 24-h period (including daytime sleeps) whilst we focused only on night-time sleep duration. Also, the earlier study assessed sleep duration in infancy (age 1–2 years) whilst we focused on sleep duration in preschool children (age 3½ years). We found the strongest association between a longer night-time sleep duration and decreased odds of daytime wetting, whilst there was weaker evidence of an association between sleep duration and bedwetting alone.

A previous study examined associations between sleep problems and both enuresis and daytime UI but did not find evidence for cross-sectional associations [10]. In agreement with this study, we did not find evidence for associations between sleep problems and an increased risk of bedwetting alone. Some previous studies have found that sleep problems are more common in children with enuresis, but these findings are based on small case–control studies of clinically referred children with enuresis [5–8]. Some of these studies did not exclude children with daytime wetting [5, 6], and some used objective measures of sleep disturbances [5, 8] which might explain the differences in findings. In contrast to the study by von Gontard and colleagues [10], we found evidence that sleep problems (e.g., night-time waking, difficulty going to sleep, getting up after being put to bed) were prospectively associated with incontinence, specifically UI classes that were characterized by daytime wetting alone, or a combination of daytime and night-time wetting at school-age.

### Potential mechanisms explaining the findings

There are several possible explanations for the observed associations between sleep duration/ problems in preschool children and UI at primary school-age. There is evidence that insufficient sleep has an impact on brain development including areas that are associated with inhibitory control [26]. Lack of inhibitory control is associated with behaviour problems such as ADHD, which are strongly associated with UI [27]. The development of voluntary control over the bladder is a complex process that involves acquiring the ability to inhibit urination, despite the sensation of bladder fullness, until it is socially appropriate to urinate [28]. Insufficient sleep could affect the development of neural circuits connecting the cerebral cortex and the bladder. Poor sleep in children is also associated with an increased risk of childhood overweight and obesity [29], which are associated with an increased risk of UI [30].

We found that preschool children who had breathing problems whilst sleeping were more likely to experience delays in attaining bladder control. The ‘delayed’ class was characterised by the presence of UI (bedwetting and daytime wetting) up to age 6, followed by a decreasing prevalence of UI up to age 9. Previous research has reported that breathing disorders are more common among children with enuresis [4]. Sleep disturbance due to breathing problems could increase arousal thresholds, which could cause children to fail to respond to signs that they need to urinate [5]. Nocturnal breathing problems are common between ages 2 and 6 years and adenotonsillar hypertrophy is often the primary cause [31]. Resolution of breathing problems through adenotonsillectomy has been found to reduce enuresis [32]. There is also evidence that breathing problems whilst sleeping might also contribute to an increased risk of daytime LUTS (urgency, frequency, urgency UI), and that adenotonsillectomy can decrease these symptoms [33]. Intermittent hypoxia, due to nocturnal sleep problems, can lead to oxidative stress, which has previously been linked to daytime LUTS [34]. Alternatively, the increased levels of daytime sleepiness and hyperactivity in children with nocturnal breathing problems [35], could lead to a loss of concentration and lack of awareness of full bladder signals.

Reverse causality could provide an alternative explanation for some of the observed associations. The prospective design enabled us to show that sleep problems are present in children before incontinence is reported by parents in their school-age children. This does not rule out the possibility that bladder problems are already present in preschool children. Overactive bladder is a common cause of UI and is caused by overactivity of the detrusor (smooth muscle fibres in the bladder wall) [36]. It is, therefore, possible that detrusor overactivity in preschool children causes arousal from sleep and explains the increased occurrence of sleep problems including difficulty going to sleep and getting up after being put to bed (associated with daytime wetting) and night-time waking (associated with persistent [day and night] wetting). UI at school-age could be a result of ongoing detrusor overactivity since the preschool period.

Another explanation is that the observed associations between sleep problems and UI could be due to common causes such as neurodevelopmental disorders. Attention deficit hyperactivity disorder (ADHD) and autism spectrum disorder (ASD) are associated with a high prevalence of sleep problems [37, 38] and incontinence (especially daytime wetting) [10]. We adjusted our analysis for preschool factors that are associated with ADHD and ASD including developmental delay, difficult child temperament, and child emotional/behaviour problems, but many of the associations between sleep problems and incontinence persisted.

The cross-sectional study by von Gontard and colleagues found evidence that children with enuresis had fewer night-time wakings than their peers who had attained night-time bladder control [10]. Consistent with this finding, we found that pre-schoolers who experienced night-time waking and those who woke up early were less likely to experience bedwetting alone at school-age. Children with enuresis are frequently regarded by their parents as deep sleepers or having difficulty waking in the morning, leading to the suggestion that they have higher arousal thresholds [11]. There is evidence, however, that children with enuresis experience sleep fragmentation and transient arousals which have been detected using polysomnography and may not be observed by parents [39]. It has also been suggested that cortical arousals, sleep fragmentation and poor-quality sleep may affect heart rate and blood pressure, which could cause excess urine production and bladder overactivity [3].

## Conclusions

The underlying aetiological mechanisms linking sleep problems and UI are yet to be elucidated and require further studies using objective measures of sleep characteristics in children with different patterns of UI. It is unclear whether sleep problems are a cause, consequence, or comorbidity of UI. Due to the heterogeneity of UI, it is likely that pathophysiological mechanisms linking sleep and UI will differ in children with bedwetting alone compared to those who suffer from both daytime and night-time wetting.

In clinical practice, sleep duration and sleep problems should be addressed in all children with enuresis and/or daytime wetting. In preschool children, sleep problems including difficulty going to sleep, getting up after being put to bed, and night-time waking could potentially identify children who will have subsequent problems attaining and maintaining daytime bladder control.

## Data Availability

ALSPAC data access is through a system of managed open access. The steps below highlight how to apply for access to the data included in this paper and all other ALSPAC data. (1) Please read the ALSPAC access policy (http://www.bristol.ac.uk/media-library/sites/alspac/documents/researchers/dataaccess/ALSPAC_Access_Policy.pdf) (PDF, 891kB) which describes the process of accessing the data and samples in detail, and outlines the costs associated with doing so. (2) You may also find it useful to browse our fully searchable research proposals database (https://proposals.epi.bristol.ac.uk/), which lists all research projects that have been approved since April 2011. (3) Please submit your research proposal for consideration by the ALSPAC Executive Committee (https://proposals.epi.bristol.ac.uk/). You will receive a response within 10 working days to advise you whether your proposal has been approved. If you have any questions about accessing data or samples, please email alspac-data@bristol.ac.uk (data) or bbl-info@bristol.ac.uk (samples).
